# Mortality and Causes of Death in Boys and Men Born With Hypospadias: A Swedish Population-Based Cohort Study

**DOI:** 10.1097/UPJ.0000000000000860

**Published:** 2025-07-15

**Authors:** Lottie Phillips, Giorgio Tettamanti, Ann Nordgren, Anna Skarin Nordenvall, Agneta Nordenskjöld

**Affiliations:** 1 Department of Women’s and Children’s Health, and Center of Molecular Medicine, Karolinska Institutet, Stockholm, Sweden; 2 Department of Urology, Södersjukhuset, Stockholm, Sweden; 3 Department of Molecular Medicine and Surgery, Karolinska Institutet, Stockholm, Sweden; 4 Unit of Epidemiology, Institute of Environmental Medicine, Karolinska Institutet, Stockholm, Sweden; 5 Department of Clinical Genetics and Genomics, Karolinska University Hospital, Stockholm, Sweden; 6 Department of Clinical Genetics and Genomics, Sahlgrenska University Hospital, Gothenburg, Sweden; 7 Institute of Biomedicine, Department of Laboratory Medicine, University of Gothenburg, Gothenburg, Sweden; 8 Department of Radiology, Karolinska University Hospital, Stockholm, Sweden; 9 Department of Pediatric Surgery, Astrid Lindgren Children’s Hospital, Karolinska University Hospital, Stockholm, Sweden

**Keywords:** hypospadias, cohort studies, mortality, cause of death

## Abstract

**Introduction:**

Although hypospadias is not a life-threatening condition, studies have found perinatal factors and comorbidities which could increase mortality. We aimed to investigate mortality and causes of death in boys and men born with hypospadias.

**Methods:**

We created a cohort of almost 3 million individuals including 16,890 with hypospadias using Swedish registers. We used Cox regression analysis to measure associations between hypospadias and all-cause mortality in different age groups (maximum age 65 years) as well as cause-specific mortality in adolescents and adults.

**Results:**

We found associations between hypospadias and mortality in infancy (HR 2.07, 95% CI: 1.74-2.45), childhood (HR 1.77, CI: 1.34-2.33), and adolescence and adulthood (HR 1.31, CI: 1.11-1.56), with stronger associations for proximal hypospadias. Controlling for birth weight and congenital comorbidity significantly reduced the association in infancy. The association was lower in younger adults (HR 1.22, CI: 1.00-1.50) but increased again after age 35 years (HR 1.57, CI: 1.17-2.11). We found a significant association with death due to cardiovascular disease or diabetes (HR 3.20, CI: 1.93-5.32) and kidney or urological disease (HR 5.16, CI: 2.13-12.5), but not cancer overall, suicide, or accidents.

**Conclusions:**

While mortality overall was low, hypospadias is associated with relatively increased mortality from infancy to middle age. In early childhood, this is related to prenatal and perinatal factors. In adolescence and adulthood, the risk of death due to cardiovascular and urological disease was increased, providing further insight into long-term health in this patient group.

Hypospadias is defined by an ectopic ventral placement of the urethral opening, with phenotypes varying from distal to proximal, found in around 1 in 125 boys born each year in Sweden.^1^ Surgery is commonly performed in childhood.^2^ While postoperative results are often successful, possible complications include urethral strictures and fistulas that can cause voiding difficulties, as well as persistent penile curvature and sexual dysfunction.^3^ Hypospadias is considered nonlife-limiting, with mostly treatable and nonsevere complications. However, it is associated with comorbidities and outcomes which may increase mortality.

**UPJ Insight**

**Study Need and Importance**
Hypospadias, a common congenital condition in men, has been linked to various comorbidities and long-term outcomes, including androgen-related disease, but its impact on mortality has not been assessed. This study aimed to investigate all-cause and cause-specific mortality from infancy to adulthood.
**What We Found**
Using Swedish national registers, the study found an increased risk of death in individuals with hypospadias, particularly those with proximal phenotypes. Increased infant and childhood mortality was largely explained by low birth weight, prematurity, and congenital comorbidities. In adolescence and adulthood, increased mortality remained after adjusting for these factors. There was a significantly elevated risk of death from cardiovascular disease and urological causes, but not from nonurological cancer, suicide, or accidents (Figure).
**Limitations**
Limitations include lack of detailed information on lifestyle and treatment factors and that the numbers of deaths in adolescents and younger adults are small, affecting statistical power even in this large population-based study. This exploratory design restricts clinical applicability for individual risk prediction.
**Interpretation for Patient Care**
Although the absolute mortality risk is low, long-term follow-up of urological and andrological health may be warranted in patients with hypospadias, particularly those with proximal forms. Further studies and clinical observation are needed to be able to more fully understand which individuals are at risk of which outcomes.

**Figure.**
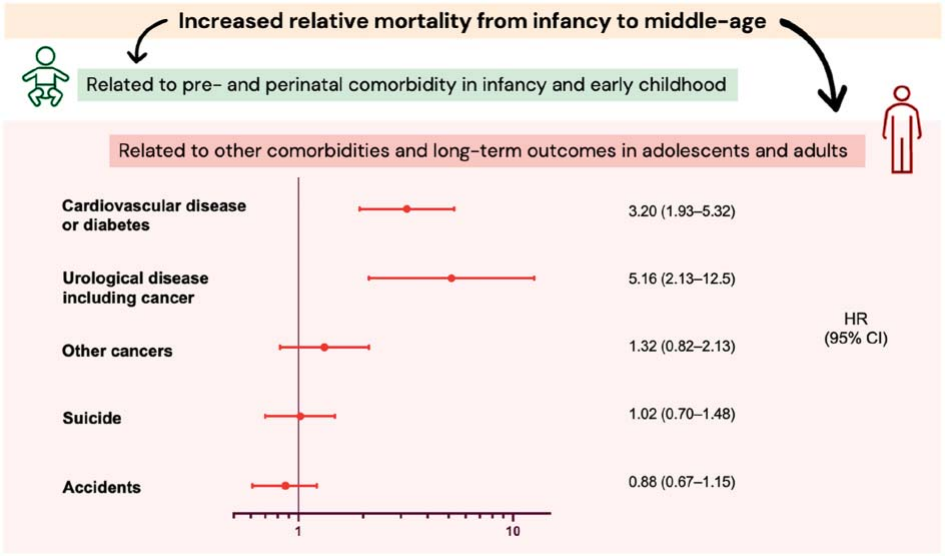
Summary of main findings. The estimated HRs for different causes of death are from age 10 to 65 years comparing men with hypospadias to unaffected men in the Swedish population born from 1954 to 2008.

First, in infancy and childhood, hypospadias is associated with pregnancy complications and congenital conditions which may be fatal. Hypospadias is strongly associated with intrauterine growth restriction (IUGR) and being born preterm and/or small for gestational age, and around 30% of those with proximal hypospadias in Sweden have a low birth weight.^4-6^ The most common co-occurrent conditions are cryptorchidism and inguinal hernia but encompass a range of both isolated and complex malformations such as heart and limb defects. Hypospadias is further overrepresented in many genetic syndromes, especially those associated with low birth weight.^5-7^

Second, recent studies have found associations with morbidities in adolescence and adulthood which can affect mortality. This includes cardiovascular disease, a leading cause of death globally.^4,8^ Some of these associations are hypothesized to relate to a higher prevalence of hypogonadism, which has in itself been linked to mortality, although it is uncertain whether the relationship is causal.^4,9^ Furthermore, possible associations have been found with forms of urological cancer which could affect mortality rates.^10^ Long-term complications of hypospadias could also cause potentially lethal outcomes including UTIs and kidney failure, but this has not been fully investigated.

Finally, some studies have addressed possible psychiatric morbidity in hypospadias, indicating an increased co-occurrence of neuropsychiatric disorders but largely similar psychosocial outcomes to the general male population.^11-14^ Neuropsychiatric conditions are linked to an increased risk of death by unnatural causes, including accidents and suicide.^15^ Studies on psychosocial outcomes after hypospadias are generally limited by methodology and have to our knowledge not explored mortality.

We aimed to use a population-based cohort to investigate mortality in individuals with hypospadias. We hypothesized that all-cause mortality would be increased across age groups, that the increase in childhood would relate to perinatal comorbidity, and that we would find increased cardiovascular and urological mortality as well as possibly increased unnatural deaths in adolescence and adulthood.

## Materials and Methods

This study is reported in accordance with the STROBE guidelines for observational studies. Ethical permission was granted by the Swedish Ethical Review Authority.

### Study Population and Exposure

We used Sweden’s national registers, linked using each permanent resident’s personal identification number.^16^ Demographic data from the Total Population Register were used to create a cohort of boys and men born 1954 to 2018.^17^

Exposure data were retrieved from the National Patient Register (NPR), started in 1964 with national coverage from 1987, and the Medical Birth Register (MBR) started in 1973.^18,19^ Individuals diagnosed with hypospadias at least once in either register were defined as exposed, while those diagnosed with both hypospadias and epispadias were excluded due to uncertain diagnosis (International Classification of Diseases [ICD] codes listed in Supplementary Table S1, https://www.urologypracticejournal.com).

**Figure 1.**
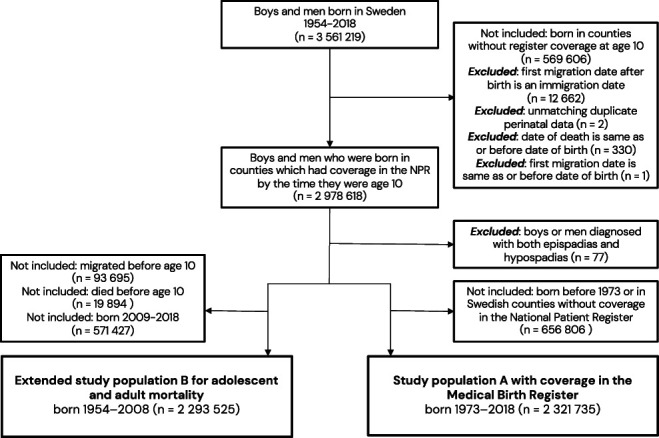
Flowchart of inclusions and exclusions for the study populations. NPR indicates National Patient Register.

Two study populations were created: Population A including individuals born from 1973 in counties with both NPR and MBR coverage from birth and Population B born 1954 to 2008 to study an older population in adolescence and adulthood (Figure 1). Data on county of birth (Total Population Register) were used to restrict population B to those born in a county which reached NPR coverage by the time the index person was 10 years to ensure more correct exposure classification.

### Outcomes and Covariates

The main outcome was all-cause mortality. All dates and causes of death 1964 to 2018 were taken from the Swedish Cause of Death Register started in 1958.^20^ We used the registered underlying cause of death to define categories based on prior research (cardiovascular disease and diabetes, urological disease including cancer, other cancers, suicides, and accidents; Supplementary Table S2, https://www.urologypracticejournal.com).

Maternal country of origin was used as a proxy for ethnicity. From the MBR, we used data on birth weight (grams) and gestational age (weeks) as continuous variables to control for perinatal factors. We used diagnostic codes from the NPR and MBR to define co-occurring congenital conditions, defined as life-limiting malformations or syndromes, as well as any extragenital malformation or syndrome (Supplementary Table S3, https://www.urologypracticejournal.com).

### Statistical Analysis

First, mortality rates and standardized mortality ratios were calculated by birth year strata in the whole study population.

Second, multiple Cox proportional hazards regression analysis was used to estimate associations between hypospadias and all-cause mortality. This was done in age brackets reflecting life periods with different potential causes of mortality highlighted in the introduction. In population A, we looked at infancy (0-<1) and childhood (1-17). In population B, we looked at age 10 to 65 as well as 10 to 34 and 35 plus to differentiate adolescence and early adulthood and middle age. Individuals who emigrated during follow-up were censored at emigration. All analyses were controlled for year of birth and maternal country of birth. Smoothed hazard estimates were assessed graphically, and Schoenfeld’s residuals were used to test for proportional hazards. Birth year showed nonproportional hazards but was included as a continuous variable to avoid issues from categorization. Stratified Cox regression was used for categorical covariates with nonproportional hazards. In population A, we also addressed perinatal and congenital factors. A Cox regression was performed controlling for birth weight and gestational age. Next, the model was adjusted for both perinatal factors and life-limiting congenital conditions. In a sensitivity analysis, we adjusted for perinatal factors and all extragenital congenital conditions. We included interaction terms in separate models to look at whether the association between mortality and hypospadias was modified by co-occurrent life-limiting congenital conditions or low birth weight (<2500 g).

Finally, we studied cause-specific mortality from age 10 years using Cox regression, censoring other causes of death at time of death. In population A (age 10-45), we looked at deaths due to unnatural causes (suicide and accidents) and natural causes (all remaining deaths), controlling for perinatal and congenital factors as described above. We studied specific causes of death in population B (age 10-65 years). The Swedish Cause of Death Register lacks specific codes for suicides and accidents before ICD-10 (Supplementary Table S2, https://www.urologypracticejournal.com). We therefore performed a sensitivity analysis from the start of ICD-10 (1997).

All analyses were performed using Stata 17.0.

## Results

### All-Cause Mortality

A total of 326 (1.8%) deaths occurred in individuals with hypospadias compared with 53,104 (1.8%) in the general population. Most childhood deaths occurred in infancy in both groups (Table 1). Mortality rates were somewhat higher in those with hypospadias, especially at younger ages, reflected in higher standardized mortality ratios in later birth years (Table 2).

**Table 1.** Characteristics of the Study Population

	No hypospadias	Any hypospadias	Distal hypospadias	Proximal hypospadias
Whole study population (A + B), born 1954-2018				
Total	2,961,320	16,890	11,850	1447
Birth year, No. (%)				
1954-1964	160,117 (5.4)	170 (1.0)	80 (0.7)	11 (0.8)
1965-1975	499,054 (17)	1532 (9.1)	1083 (9.1)	75 (5.2)
1976-1986	539,007 (18)	2456 (15)	1849 (16)	104 (7.2)
1987-1997	625,421 (21)	3465 (21)	1435 (12)	240 (17)
1998-2008	553,150 (19)	4369 (26)	3538 (30)	519 (36)
2009-2018	584,571 (20)	4898 (29)	3865 (33)	498 (34)
Mother’s country of birth, No. (%)				
Nordic country (including Sweden)	2,591,350 (88)	13,577 (80)	9420 (80)	1109 (77)
Greater Europe	147,748 (5.0)	1251 (7.4)	893 (7.5)	99 (6.8)
Africa	51,275 (1.7)	529 (3.1)	394 (3.3)	80 (5.5)
Asia	133,757 (4.5)	1367 (8.1)	1033 (8.7)	144 (10)
Other	32,057 (1.1)	157 (0.9)	109 (0.9)	15 (1.0)
Missing	5133 (0.2)	9 (0.1)	NA	NA
Death at any age 0-65, No. (%)	53,104 (1.8)	326 (1.8)	162 (1.4)	34 (2.4)
Age 10-65	33,800 (1.1)	136 (0.8)	82 (0.7)	12 (0.8)
Age 10-34	19,903 (0.7)	92 (0.5)	49 (0.4)	9 (0.6)
Age 35-65	17,536 (0.6)	58 (0.3)	42 (0.4)	NA
Study population A, born 1973-2018				
Total	2,302,125	15,229	10,721	1360
Death at any age 0-45, No. (%)	26,043 (1.1)	243 (1.6)	104 (1.0)	29 (2.1)
Age <1	10,896 (0.5)	134 (0.9)	42 (0.4)	17 (1.3)
Age 1-17	5137 (0.2)	52 (0.3)	27 (0.3)	8 (0.6)
Age 10-45	12,194 (0.5)	74 (0.5)	39 (0.4)	8 (0.6)
Any extragenital malformation or chromosome aberration, No. (%)	211,005 (9.2)	3241 (21)	2036 (19)	539 (40)
Life-limiting congenital condition, No. (%)	25,122 (1.1)	629 (4.1)	333 (3.1)	152 (11)
Gestational week, No. (%)				
≤31	19,589 (0.9)	498 (3.3)	266 (2.5)	143 (11)
32-36	117,955 (5.1)	1556 (10)	984 (9.2)	233 (17)
37-42	2,097,162 (91)	12,791 (84)	9203 (86)	938 (69)
≥43	18,222 (0.8)	101 (0.7)	74 (0.7)	5 (0.4)
Missing	49,197 (2.1)	283 (1.9)	194 (1.8)	41 (3.0)
Birth weight, No. (%), g				
<2500	88,592 (3.9)	1947 (13)	1105 (10)	435 (32)
2500-4500	2,064,592 (90)	12,530 (82)	9072 (85)	847 (62)
>4500	97,637 (4.2)	448 (2.9)	342 (3.2)	26 (1.9)
Missing	51,304 (2.2)	304 (2.0)	202 (1.9)	52 (3.8)

Abbreviations: NA, not applicable.

Unadjusted total prevalence of exposure, covariates, and deaths in the study population. If n < 5, numbers are not presented and instead marked with NA.

**Table 2.** Mortality Rates and Standardized Mortality Ratios

Year of birth	No hypospadias	Any hypospadias
1954-1975		
Total N (mortality rate per 10,000 person y)	27,588 (8.9)	78 (10)
SMR (95% CI)		1.14 (0.91-1.42)
1976-1997		
Total N (mortality rate per 10,000 person y)	21,076 (6.1)	164 (9.5)
SMR (95% CI)		1.56 (1.34-1.82)
1998-2018		
Total N (mortality rate per 10,000 person y)	4440 (4.0)	84 (9.5)
SMR (95% CI)		2.34 (1.89-2.90)

Abbreviations: SMR, standardized mortality ratio.

Standardized mortality rates per 10,000 person years in strata of birth years. Standardized mortality ratios are calculated against the rates within calendar year strata for the full study population. They do not take into consideration age at time of death or potential confounding.

**Figure 2.**
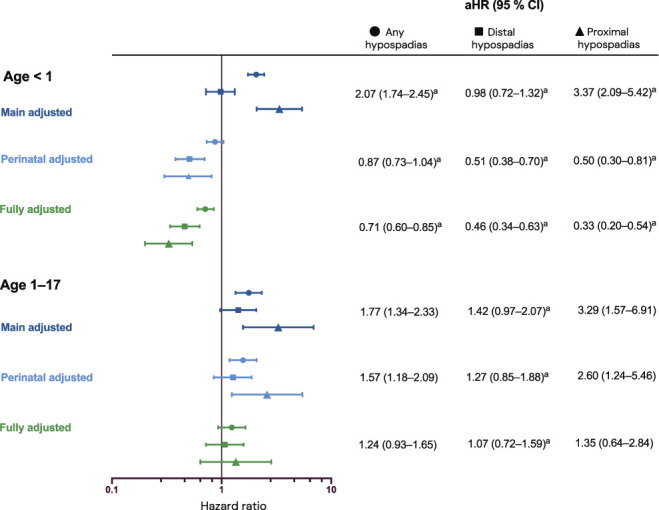
All-cause mortality in children born with hypospadias from 1973. Population A born from 1973 to 2018 in counties that had coverage in the National Patient Register and Medical Birth Register from birth. Followed between the ages (years) listed to the left or until whichever occurred first of death, migration, or the end of follow-up on December 31, 2018. Attained age was the underlying timescale. Main adjusted (dark blue) results are adjusted for year of birth and maternal country of birth. Perinatal adjusted (light blue) is further adjusted for birth weight and gestational age. Fully adjusted (green) is also adjusted for life-limiting congenital conditions (Supplementary Table S3, https://www.urologypracticejournal.com). ^a^Hazards are not proportional across the follow-up time. aHR indicates adjusted hazard ratio.

In population A (born 1973-2018), we identified 2,321,735 individuals (Figure 1), including 15,229 with hypospadias (Table 1). We estimated an increased risk of dying in infancy (HR 2.07, 95% CI: 1.74-2.45) and childhood (HR 1.77, 95% CI: 1.34-2.33). The associations were stronger for proximal hypospadias. We found no association between distal hypospadias and infant mortality. Adjusting for birth weight and gestational age reduced estimates to below the null in infancy. In childhood, the results were reduced toward the null and no longer statistically significant. Additional adjustment for life-limiting congenital conditions further decreased effect estimates somewhat (Figure 2, Supplementary Table S4, https://www.urologypracticejournal.com), with similar results adjusting instead for extragenital congenital comorbidities (Supplementary Table S5, https://www.urologypracticejournal.com). We found no significant interaction between hypospadias and life-limiting congenital comorbidity. Mortality was higher among low birth weight infants, especially those without hypospadias (Supplementary Table S6, https://www.urologypracticejournal.com). Among infants, the median age at the time of death was lower in those with low birth weight compared with those of normal-high birth weight (21 and 34 days, respectively) and higher among those with hypospadias (47 days).

**Figure 3.**
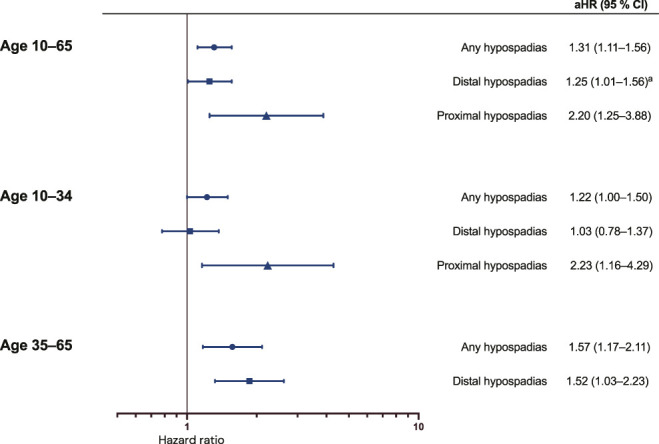
All-cause mortality in adolescents and adults born with hypospadias from 1954. Population B born from 1954 to 2008 in counties that had coverage in the National Patient Register by the time they were age 10 years. Followed between the ages (years) listed to the left or until whichever occurred first of death, migration, or the end of follow-up on December 31, 2018. Attained age was the underlying timescale. Results are adjusted for year of birth and maternal country of birth. Proximal hypospadias for age 35 to 65 years not included due to too small numbers (n < 5). ^a^Hazards are not proportional across the follow-up time. aHR indicates adjusted hazard ratio.

In population B born from 1954, including 2,293,525 individuals, hypospadias was somewhat associated with increased mortality in adolescence/early adulthood (HR 1.22, 95% CI: 1.00-1.50) and more strongly in middle age (HR 1.57, 95% CI: 1.17-2.11). Associations were larger for proximal hypospadias; distal hypospadias was only associated with mortality in middle age (Figure 3, Supplementary Table S7, https://www.urologypracticejournal.com).

### Causes of Death in Adolescence and Adulthood

**Figure 4.**
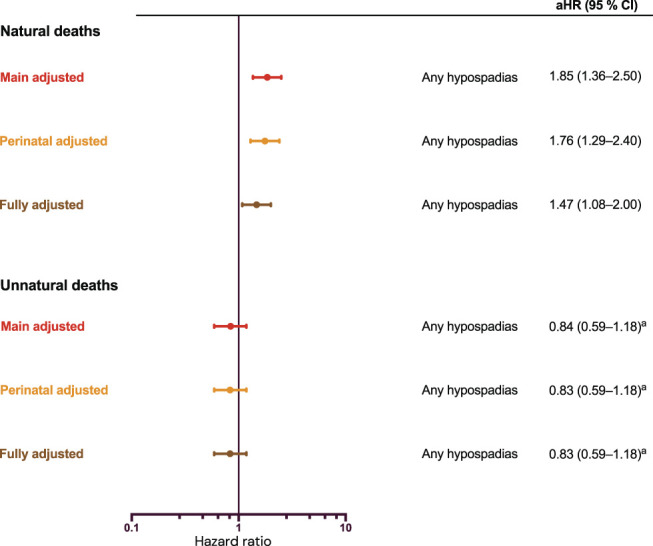
Mortality due to natural and unnatural causes in adolescents and adults born with hypospadias from 1973. Population A included individuals born from 1973 to 2008 in counties that had coverage in the National Patient Register and Medical Birth Register from birth. Followed from age 10 until whichever occurred first of death, migration, or the end of follow-up on December 31, 2018. Attained age was the underlying timescale. Main adjusted (red) results are adjusted for year of birth and maternal country of birth. Perinatal adjusted (orange) is further adjusted for birth weight and gestational age. Fully adjusted (brown) is also adjusted for life-limiting congenital conditions (Supplementary Table S3, https://www.urologypracticejournal.com). ^a^Hazards are not proportional across the follow-up time. aHR indicates adjusted hazard ratio.

In population A, unnatural deaths due to accidents or suicide were dominating, but the proportion was lower among those with hypospadias than without (41% and 64% respectively). We found an association with natural deaths (HR 1.85, 95% CI: 1.36-2.50) even after adjusting for perinatal factors and congenital comorbidity (HR 1.47, 95% CI: 1.08-2.00), but no association with unnatural deaths (Figure 4).

**Figure 5.**
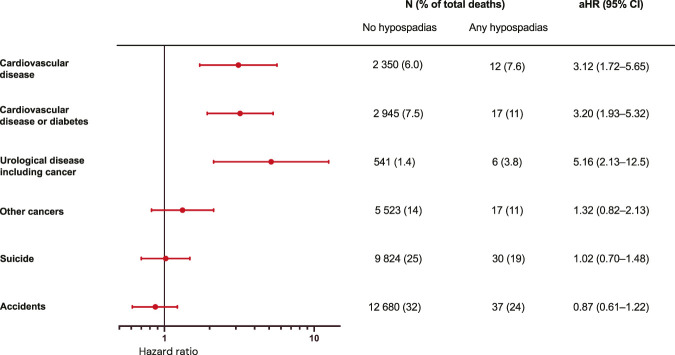
Cause-specific mortality in adolescents and adults born from 1954. Population B born from 1954 to 2008 in counties that had coverage in the National Patient Register by the time they were age 10 years. Followed from age 10 until whichever occurred first of death, migration, or the end of follow-up December on 31, 2018. All individuals who died of other causes than the cause studied were censored at time of death. Attained age was the underlying timescale. Results are adjusted for year of birth and maternal country of birth. aHR indicates adjusted hazard ratio.

In population B, there were relatively more cardiovascular deaths (9% and 5.9%, respectively) and fewer deaths due to nonurological cancer (9% and 14%, respectively) in the hypospadias group compared with the general population. We found a 3-fold increased risk of dying from cardiovascular disease or diabetes (HR 3.20, 95% CI: 1.93-5.32) and a 5-fold increased risk of dying from urological disease including cancer (HR 5.16, 95% CI: 2.13-12.5), but no association with dying from cancer overall, suicide, or accidents (Figure 5, Supplementary Table S8, https://www.urologypracticejournal.com). The 6 deaths from urological disease included urological cancer, kidney failure, and infection. Looking from the start of ICD-10, we still did not find an increased risk of dying from suicide (HR 0.88, 95% CI: 0.39-1.95) or accidents (HR 0.54, 95% CI: 0.22-1.30).

## Discussion

In this nationwide Swedish study following men until age 65 years using register data, we found an association between hypospadias and mortality across age groups, with stronger associations in infancy and middle age. Low birth weight, preterm birth, and congenital comorbidity were driving the associations in infancy and to a lesser extent childhood, while adult mortality appears to be more related to other factors. Looking at cause-specific mortality, we found an association with death from cardiovascular and urological disease including cancer, but not cancer overall, suicide, or accidents.

As in previous studies, those with proximal hypospadias have a particularly large increase in risk compared with the general population. Distal hypospadias was not associated with increased mortality in infancy, potentially due to a smaller association with perinatal morbidity. However, relative mortality in distal hypospadias increased in adulthood, indicating more long-term risks.

Our findings support that mortality in early life in those with hypospadias is increased due to prenatal and perinatal factors, including IUGR and comorbidity with genetic syndromes or other malformations. Being born preterm or with low birth weight is heavily associated with infant and child mortality but also long-term disease including cardiovascular and metabolic disease.^21,22^ However, we found that the association with mortality from natural causes in adolescents and adults remained after adjustment, indicating that causes other than IUGR play a larger role later in life in those with hypospadias.

Adjusting for low birth weight, hypospadias appeared protective against infant mortality. However, as the median age at death in low birth weight infants was 21 days, there are likely cases where a hypospadias diagnosis was not registered before death. This is supported by the higher median age at death (before 1 year) among those with hypospadias. Those with the lowest birth weight and subsequent early mortality will be defined as not having hypospadias, causing a false protective association.

Our results on specific causes of death support previous findings of increased cardiovascular and urological morbidity in adolescents and adults with hypospadias and highlight the importance of access to long-term follow-up.^4,8,10^ Previous research in this study cohort also found elevated risks of both hypogonadism and cardiometabolic disease. In those analyses, we found that the risk of cardiovascular disease could not be fully explained by shared familial factors or perinatal comorbidity, consistent with our finding of persistent adult mortality risk after adjustment. Although androgen dysfunction has been suggested as a cause,^4,8^ there may be other mediating factors such as smoking and lifestyle which we lacked data on. However, current evidence supports clinical vigilance for hypogonadism in those with proximal hypospadias especially. The observed increased risk of dying from urological disease should be interpreted cautiously and is not yet clinically applicable given the small number of deaths and broad diagnoses categories. As a population-based study, we did not include detailed information on individual deaths due to ethical and legal constraints. We strongly encourage further studies on long-term urological outcomes of hypospadias. We could not demonstrate an association with dying from nonurological cancer in adolescence and adulthood, contrary to prior findings indicating a link between malformations and cancer overall.^23,24^ Our results on dying from suicide and accidents are encouraging, indicating an absence of significant psychosocial differences leading to increased mortality from unnatural causes in these age groups. However, this does not mean that no individuals with hypospadias face significant struggles that would benefit from further support.

The major strength of this study is our national registers allowing for a population-based cohort study over an extended period. The Swedish Cause of Death Register is highly complete and accurate, especially for those 64 years and younger (>90% accuracy for cause of death).^20^ A further strength is that we were able to address different causes of increased mortality by controlling for prenatal and perinatal factors and looking at specific causes of death hypothesized to be linked to hypospadias.

There is a possible issue with depletion of susceptibles given higher childhood mortality in those with hypospadias. The absolute risk of childhood death was very low and unlikely to affect later all-cause mortality. However, this may play a role for cause-specific mortality, especially causes strongly linked to age, such as many types of cancer.

Our results are likely generalizable to other populations, especially with similar health profiles, but our younger cohort prevents drawing conclusions about mortality in older men.

Finally, this study is exploratory in nature, aiming to shed more light on long-term risks indicated by previous studies, but we cannot yet fully differentiate which individuals with hypospadias are at risk of early mortality or why. Therefore, our findings are not immediately clinically applicable but lend more information to our understanding of hypospadias and warrant further observation. There are possible mediators for increased mortality, including testosterone levels, impact of certain or repeated complications and treatments, specific genetic variants causing morbidity, and lifestyle factors, which should be explored further.

## Conclusions

In this cohort study using Swedish registers, we have found an association between hypospadias and increased mortality in childhood, adolescence, and adulthood, although absolute mortality risk was low. Increased infant mortality, and to a lesser extent childhood mortality, was explained by being born with low birth weight, preterm, or with congenital comorbidity, as we could not demonstrate an association in adjusted models. However, in adolescents and adults, other factors such as androgen-related comorbidity and long-term hypospadias complications likely play a larger role. Our findings for cause-specific mortality support prior research indicating associations with cardiovascular disease and urological cancer, while we could not demonstrate any association with overall cancer mortality, suicides, or lethal accidents. This provides insight into long-term health risks which warrant further observation through long-term follow-up including clinical, epidemiological, and molecular or mechanistic studies.

## Supplementary Material

SUPPLEMENTARY MATERIAL
