# Supplementary material for: Mortality and Causes of Death in Boys and Men Born With Hypospadias: A Swedish Population-Based Cohort Study
Source: Urol Pract. 2025 Jul 15;12(6):733–42. doi: 10.1097/UPJ.0000000000000860 (PMC12551417; doi:10.1097/UPJ.0000000000000860)
Supplement: SUPPLEMENTARY MATERIAL [file urpr-12-733-s001.pdf]

Supplementary material

Table S1 ICD codes for hypospadias and epispadias

**ICD-7**<br>1958–1968	**ICD-8**<br>1969–1986	**ICD-9**<br>1987-1996	**ICD-10**<br>1997-
	**Hypospadias**		
Hypospadias (757.21)	Hypospadias glandis (752.20)[a]	Hypospadias (752G)	Glandular hypospadias (Q54.0)[a]
	Hypospadias scrotalis (752.21)[b]		Penile hypospadias (Q54.1)[a]
	Hypospadias totalis (752.22)[b]		Penoscrotal hypospadias (Q54.2)[b]
	Hypospadias alia sive NUD (752.29)		Perineal hypospadias (Q54.3)[b]
			Hypospadias not otherwise specified (Q54.8, Q54.9)
	**Epispadias**		
757.20	752.30	[c]	Q64.0

[a]Grouped as distal hypospadias.
[b]Grouped as proximal hypospadias.
[c]In ICD-9, epispadias and hypospadias have the same ICD code (752G). We defined all men with that diagnosis as having hypospadias and excluded everyone who had also received a specific epispadias diagnosis in ICD-7, ICD-8, or ICD-10.

Table S2 ICD-codes for cause-specific mortality.

	ICD-7	ICD-8	ICD-9	ICD-10
**Cardiovascular Disease**[a]	400.10, 400.30, 401-404, 410, 412-413, 424.1, 427.0, 427.91, 427.92, 432, 433, 437, 435, 440, 441	400.10, 400.30, 401-404, 410, 412-413, 424.1, 427.0, 427.91, 427.92, 432, 433, 437, 435, 440, 441	401-404, 410, 411B, 412-414, 4241, 4271, 4273, 4274, 428, 433, 434, 4370-4371, 435, 440, 441	I10-I13, I20-I22, I25, I35, I47.2, I48, I49.0, I50, I63, I65, I66, I70, I71, G45
**Diabetes**[a]	260	250	250	E10, E11, E13, E14
**Urological disease**	59-61	58-60	58-60	N0-N5, N99
**Urological cancer**	170, 177-179, 1800, 1801, 1809, 1810, 1811, 1812, 1816-1819, 1890, 1896	174, 1859, 1869, 1725, 1735, 187-189	174, 1859, 1869, 187–189	C50, C60-C67, C80, C81, C688, C689
**Any cancer**[b]	14-20	14-20	15-20	C
**Suicide**[c]	97, 963	95, 98	95, 98	X6, X7, X80-X85, Y1 -Y34
**Accidents**[c]	80-86, 88-93, 960, 961, 962	80-93, 98	80-86, 88-92, 98	V, W, X0-X5, Y1-Y34, Y85-Y86, Y89

[a]Same codes as used previously to study androgen-related outcomes in adolescent and men born with hypospadias.[1]
[b]All individuals with an underlying cause of death that is listed under "any urological disease" were redefined as not having died of cancer in the analyses for "other cancer".
[c]Overlap in codes between accidents and suicide reflect codes for when it is unclear whether the death was accidental or not.

	ICD-8	ICD-9	ICD-10
**All**	74-75	74-75, 237H	All Q
**DSD-related conditions**	752, 759.51-759.53	752, 758G, 758H, 758W, 758X	Q5, Q97, Q98, Q99.0, Q99.1
**Life-limiting conditions**	740, 74100, 74200, 74300, 74399, 7460, 7462, 7463, 7465, 7466, 7467, 7473, 7474, 7475, 7483, 7485, 7486, 7488, 7508, 75110, 75152, 75161, 75162, 75300, 7558, 75558, 75598, 75600, 7564, 7565, 7572, 7594, 7598	7400, 7410, 7420, 7422, 7423, 7424, 7450, 7451, 7452, 7453, 7459, 7460, 7461, 7462, 7463, 7465, 7466, 7467, 7468, 7469, 7472, 7473, 7474, 7478, 7483, 7485, 7486, 7488, 7489, 7504, 7511, 7515, 7516, 7530, 7548, 7555, 7558, 7560, 7564, 7565, 7567, 7571, 7573, 7581, 7582, 7584, 7585, 7586, 7596, 7598	Q00.0 Q01 Q03.1 Q03.9 Q04.0 Q04.4 Q04.6 Q04.9 Q07.0 Q20.0 Q20.3 Q20.4 Q20.6 Q20.8 Q21.3 Q21.9 Q22.0 Q22.1 Q22.4 Q22.5 Q22.6 Q23.0 Q23.2 Q23.4 Q23.9 Q25.4 Q25.6 Q26.2 Q26.4 Q26.8 Q28.2 Q32.1 Q33.6 Q34 Q39.6 Q41.0 Q43.7 Q44.2 Q44.5 Q44.7 Q60.1 Q60.6 Q61.9 Q64.2 Q74.3 Q74.8 Q75.0 Q77.2 Q77.3 Q77.4 Q78.0 Q78.5 Q79.2 Q79.3 Q80.4 Q81 Q82.1 Q85.8 Q86.0 Q87.0 Q87.1 Q87.8 Q91-Q95

Table S4 Unadjusted estimates for all-cause mortality in study population A born 1973 to 2018

	Unadjusted HR (95% CI)		
	**Any hypospadias**	**Distal hypospadias**	**Proximal hypospadias**
**0–1**	1.86 (1.57–2.21)[a]	0.85 (0.63–1.14)[a]	2.65 (1.65–4.26)[a]
**1–17**	1.67 (1.27–2.20)	1.31 (0.90–1.92)[a]	2.75 (1.31–5.76)

[a] Hazards are not proportional across the follow-up time. The exact hazard ratio should be interpreted with caution and focus put on the confident intervals.

Table S5 All-cause mortality in study population A born 1973 to 2018: sensitivity analysis adjusted for all extragenital malformations

	Unadjusted HR (95% CI)		
	**Any hypospadias**	**Distal hypospadias**	**Proximal hypospadias**
**0–1**	0.72 (0.61–0.87)[a]	0.43 (0.32–0.59)[a]	0.39 (0.24–0.63)[a]
**1–17**	1.27 (0.95–1.69)	1.05 (0.71–1.56)[a]	1.71 (0.81–3.59)

[a] Hazards are not proportional across the follow-up time. The exact hazard ratio should be interpreted with caution and focus put on the confident intervals.

Table S6 Interaction analysis for life-limiting congenital comorbidity and birth weight in study population A born 1973-2018

		Adjusted HR for mortality (95% CI)		
		**No hypospadias**	**Hypospadias**	**P-value for interaction**
**Age 0-1**	**No comorbidity**	1	1.53 (1.23–1.91)	
	**Comorbidity**	21.7 (20.7–22.8)	17.1 (12.2–24.2)	0.18
	**Normal-high birth weight**	1	1.88 (1.46–2.43)	
	**Low birth weight**	22.0 (21.2–22.8)	9.05 (6.44–12.7)	0.000
**Age 1-17**	**No comorbidity**	1	1.26 (0.88–1.79)	
	**Comorbidity**	13.2 (12.1–14.4)	16.4 (9.32–28.7)	0.46
	**Normal-high birth weight**	1	1.48 (1.06–2.05)	
	**Low birth weight**	2.27 (2.05–2.51)	3.22 (1.77–5.89)	0.26
**Age 10-45**	**No comorbidity**	1	1.21 (1.01–1.45)	
	**Comorbidity**	4.31 (4.04–4.60)	4.42 (2.69–7.25)	0.93
	**Normal-high birth weight**	1	1.30 (1.08–1.54)	
	**Low birth weight**	1.46 (1.36–1.56)	1.42 (0.80–2.51)	0.92

Comorbidity refers to life-limiting congenital comorbidity (Table S3). Each analysis is adjusted for birth year and maternal country of birth. Low birth weight was defined as <2500 g.

Table S7 Unadjusted estimates for all-cause mortality in study population B born 1954-2008

	Unadjusted HR (95% CI)		
	**Any hypospadias**	**Distal hypospadias**	**Proximal hypospadias**
**10–65**	1.25 (1.05–1.48)	1.19 (0.96–1.48)[a]	2.06 (1.17–3.63)
**10–34**	1.16 (0.94–1.42)	0.99 (0.75–1.31)	2.07 (1.08–3.98)
**35–65**	1.49 (1.11–2.01)	1.73 (1.23–2.44)	NA

[a] Hazards are not proportional across the follow-up time. The exact hazard ratio should be interpreted with caution and focus put on the confident intervals.

Table S8 Unadjusted estimates for cause specific mortality in study population B born 1954-2008

	Unadjusted HR (95% CI) for any hypospadias
**Cardiovascular**	2.77 (1.53–5.02)
**Cardiovascular and diabetes**	2.90 (1.74–4.81)
**Any urological**	4.68 (1.93–11.3)
**Other cancer**	1.25 (0.78–2.01)
**Suicide**	1.01 (0.69–1.46)
**Accidents**	0.81 (0.57–1.13)

References

[1] Phillips L, Lundholm C, Kvist U, Almqvist C, Nordenskjöld A, Skarin Nordenvall A. Increased androgen-related comorbidity in adolescents and adults born with hypospadias: A population-based study. Andrology 2022;10:1376–86. https://doi.org/10.1111/andr.13229.

[2] Fraser LK, Miller M, Hain R, Norman P, Aldridge J, McKinney PA, et al. Rising national prevalence of life-limiting conditions in children in England. Pediatrics 2012;129. https://doi.org/10.1542/peds.2011-2846.
